# Hemozoin: a waste product after heme detoxification?

**DOI:** 10.1186/s13071-025-06699-x

**Published:** 2025-03-04

**Authors:** Jun Sun, Chuantao Fang, Xixi Qin, Wenwen Si, Fei Wang, Yanna Li, Xiaoli Yan

**Affiliations:** 1 School of Medicine, Tongji University, 500 Zhennan Road, Shanghai, 200331 People’s Republic of China; 2 Shanghai Tenth People’s Hospital, Tenth peoples hospital of Tongji university, Shanghai, People’s Republic of China, Shanghai, China

**Keywords:** Hemozoin, Malarial parasites, Transmission electron microscopy, Iron, Heme

## Abstract

**Background:**

Hemozoin is considered a waste byproduct of heme detoxification following hemoglobin digestion; consequently, the biological functions of hemozoin in hemozoin-producing organisms have often been overlooked. However, recent findings indicate that *Schistosoma *hemozoin facilitates the transfer of iron from erythrocytes to eggs through its formation and degradation, thereby increasing interest in the role of malarial hemozoin.

**Methods:**

Using transmission electron microscopy, we compared the formation of *Schistosoma *hemozoin and malaria hemozoin. Through transcriptome analysis of different stages of *P. falciparum* 3D7^WT^ and *P. falciparum* 3D7^C580Y^,- where the latter serves as a control with reduced hemozoin production, -we analyzed expression patterns of genes related to DNA synthesis, iron, and heme utilization. Using light microscopy, we observed hemozoin aggregation following artemether treatment, and macrophage morphology after ingesting hemozoin in vivo and in vitro.

**Results:**

Similar to *Schistosoma* hemozoin, malaria hemozoin consists of heme aggregation and a lipid matrix, likely involved in lipid processing and the utilization of heme and iron. Transcriptome analysis reveals that during the trophozoite stage, the expression levels of these genes in *P. falciparum* 3D7^WT^ and *P. falciparum* 3D7*C580Y* are higher than those during the schizont stage. Correspondingly, less hemozoin was detected at the trophozoite stage, while more was observed during the schizont stage. These results suggest that when more heme and iron are utilized, less heme is available for hemozoin formation. Conversely, when less heme and iron are utilized, they can accumulate for hemozoin formation during the schizont stage, likely benefiting lipid remodeling. Disruption of heme utilization and hemozoin aggregation may lead to parasite death. In addition, the hemozoin released by schizonts can impair macrophage functions, potentially protecting merozoites from phagocytosis. Furthermore, it may be carried by gametocytes into the next host, fulfilling their requirements for iron and heme during their development in mosquitoes.

**Conclusions:**

Hemozoin is not a waste byproduct of heme detoxification but instead plays a crucial role in the parasite’s life cycle

**Graphical Abstract:**

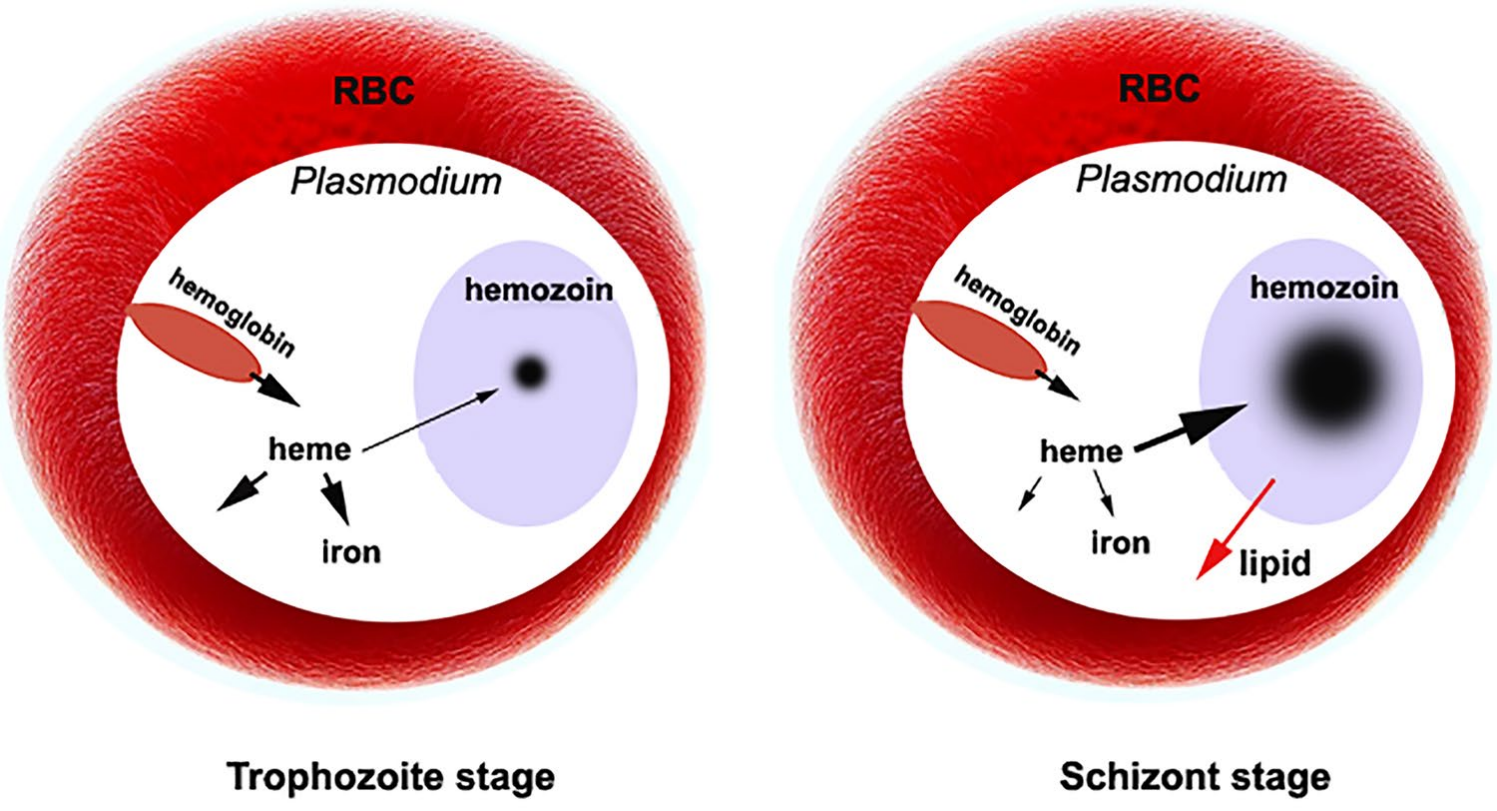

**Supplementary Information:**

The online version contains supplementary material available at 10.1186/s13071-025-06699-x.

## Background

Hemozoin is regarded as a critical byproduct produced during the unique detoxification process of malaria parasites. During the blood stages of the *Plasmodium* species, hemozoin is synthesized as the parasite digests host erythrocyte hemoglobin to obtain its amino acids. The liberated toxic heme, a compound that contains an iron atom within a porphyrin ring, is converted into an insoluble and chemically inert form to prevent cellular damage caused by its pro-oxidant properties. This substance, composed of heme polymers linked by iron–carboxylate bonds, accumulates in the parasite’s food vacuole and is subsequently released into circulation when infected red blood cells rupture.

The impact of heme on parasites is a topic of significant interest within the scientific community. Some researchers have proposed that heme can inhibit various enzymes in *Plasmodium*, including plasmepsins, falcipains, glycolytic glyceraldehyde-3-phosphate dehydrogenase, and 6-phosphogluconate dehydrogenase [1–3]. Furthermore, the incorporation of lipophilic heme into biological membranes decreases the deformability of erythrocytes and induces hemolysis. Heme within the membrane also destabilizes the lipid bilayer, rendering it more vulnerable to H_2_O_2_-mediated lysis. This integration disrupts the normal dynamic interactions between the red blood cell (RBC) membrane and its underlying cytoskeletal proteins [4]. High concentrations of heme lead to increased oxidative stress, and the presence of peroxidation products is associated with decreased RBC membrane fluidity, which likely increases cellular rigidity in parasitized red cells [5]. The catabolism of hemoglobin, which releases reactive heme and iron, is linked to the generation of redox-active substances such as H_2_O_2_, superoxide radicals, and hydroxyl radicals, all of which directly mediate lipid peroxidation [6]. The interaction among heme, intracellular hydrogen peroxide, and lipids can result in lipid peroxidation [7]. These detrimental effects can be directly and indirectly attributed to free heme [8], suggesting that hemozoin formation serves as a detoxification mechanism. Notably, beyond detoxification, hemozoin formation is likely to serve multiple functions in parasites, including acting as a beneficial metabolite and biological cofactor [9–11], stimulating the inflammasome pathway by inducing oxidative stress, and activating endothelial cells [12–15].

The malaria parasite maintains a consistent heme pool at approximately 1.6 µM throughout its development within red blood cells [16], underscoring the critical role of high heme concentrations for the parasite. While the parasite digests up to 65% of the host cell’s hemoglobin, it only utilizes about 16% of the amino acids derived from this digestion, raising the question of whether hemoglobin digestion primarily serves to acquire amino acids [17]. The effectiveness of iron chelators in eliminating malarial parasites [18–22] strongly indicates the necessity of iron for these organisms. Notably, in malaria parasites, iron is released during heme degradation, a process facilitated by glutathione (GSH) [23], suggesting that the accumulation of significant amounts of heme likely corresponds to a substantial demand for iron. Furthermore, the considerable quantities of hemozoin formed in gametocytes, and subsequently transferred to mosquitoes, implies that hemozoin plays a vital role in the storage and utilization of heme and iron, especially as malaria parasites initiate heme synthesis exclusively in mosquitoes [24, 25].

In addition, lipids also play a crucial role in *Plasmodium*, with distinct changes in lipid composition occurring at various developmental stages [26]. Notably, the parasite is unable to synthesize cholesterol de novo, and has a limited capacity for fatty acid synthesis [27, 28]. Consequently, the parasites must acquire and utilize host lipids. In the digestive vacuoles of *Plasmodium*, hemozoin formation is enhanced by host unsaturated fatty acids, while the electrons released during heme polymerization likely oxidize these unsaturated fatty acids. The remodeled fatty acids or lipids are probably detoxified and rendered suitable for use by *Plasmodium* [29, 30]. Furthermore, hemozoin-producing schistosomes have been shown to utilize hemozoin formation and degradation to transfer iron and lipids to the vitelline gland and eggs [31–33]. This evidence suggests that malarial hemozoin formation is not merely a byproduct of heme detoxification but likely plays a vital role in the parasite’s life cycle. To further investigate the role of hemozoin, we employed transmission electron microscopy (TEM) to compare the formation of hemozoin in *Schistosoma* and *Plasmodium*. Notably, malarial hemozoin was directly observed without the conventional electron microscopy sampling process, aiming to preserve the sample’s authentic structure and examine its association with the parasites. Furthermore, the K13 mutation decreases the digestion of hemoglobin in *P. falciparum*, resulting in a reduced requirement for heme or iron. Consequently, *P. falciparum* 3D7^C580Y^ is characterized by low heme or iron utilization. To investigate the relationship between iron or heme utilization and hemozoin production, differential gene expression between *P. falciparum* 3D7^WT^ and *P. falciparum* 3D7^C580Y^ was analyzed in this study.

## Methods

### Parasites and isolation of *Schistosoma* hemozoin granules (SHGs)

Female mice of the Kunming strain, weighing between 20 and 22 g, were obtained from SLRC Laboratory Animal Co., Ltd. in Shanghai, China. Cercariae freshly shed by snails were used to infect the mice percutaneously, with each mouse receiving 20 cercariae. Adult female schistosomes were collected 42 to 45 days post-infection and washed with sterile 0.15 mol/L NaCl solution (normal saline). The female schistosomes were then cut into small sections in sterile normal saline solution within Eppendorf tubes, and the resulting dark suspensions were collected, as described in a previous study [32]. Clumps were removed, and individual hemozoin granules were retained through low-speed centrifugation. The hemozoin granules were subsequently washed with sterile normal saline solution and used for a macrophage phagocytosis experiment.

An intact female adult worm was divided into three segments, and the gut contents from each segment were collected and processed according to a previously described method [31, 32]. The processed samples were examined using light microscopy (LM, Nikon 50i) and a JEOL EW-1230 scanning electron microscope at an accelerating voltage of 80 kV. Images were captured using a digital photo-documentation system (Gatan Bioscan Camera, model 792).

### Malaria parasites and hemozoin analysis with LM and TEM

The *P. falciparum* parasites (3D7 strain) were obtained from the National Institute of Parasitic Diseases, Chinese Center for Disease Control and Prevention. The *P. falciparum* 3D7^WT^ and *P. falciparum* 3D7^C580Y^ strains were cultured in RPMI 1640 medium supplemented with 25 mmol/L HEPES, 0.5% AlbuMAX, 0.2% sodium bicarbonate, 0.2 mmol/L hypoxanthine, and 20 μg/mL gentamicin sulfate in a 37 ℃ incubator with 5% CO_2_ and 5% O_2_. Fresh O+ human red blood cells (RBCs) from healthy donors were used to maintain parasitemia, and the parasites were kept at a 2% hematocrit, as reported previously [34]. Blood smears were stained with Giemsa according to standard staining protocols and subsequently examined using a light microscope (Nikon 50i).

Erythrocytes infected by malaria parasites were treated with saponin at a final concentration of 0.15% and subsequently centrifuged at 10,000 rpm for 5 min. The resulting black deposit was collected. For samples intended for lipid composition analysis, they were treated with 1–2.5% SDS for 0.5–1 h at 37 ℃. Subsequently, these samples were fixed with 2.5% glutaraldehyde in phosphate-buffered saline (PBS) (pH 7.2) and 1% osmium tetroxide, along with other samples. Subsequently, the samples were washed with PBS and dripped on copper slot grids coated with Formvar, then dried at room temperature for observation. These deposits were directly observed using a JEOL EW-1230 transmission electron microscope (TEM) (Japan) at an accelerating voltage of 80 kV. Other samples were fixed in glutaraldehyde overnight at 4 °C, washed with PBS, and then fixed in 1% osmium tetroxide. They were dehydrated in acetone and embedded in Epon 812. Thin sections, 60 nm in thickness, were cut using an ultramicrotome (EM UC6, Leica, Germany) and mounted on copper slot grids coated with Formvar. The sections were stained with uranyl acetate and lead citrate for examination. Samples were observed using a JEOL EW-1230 TEM (Japan), and images were acquired with a digital photodocumentation system (Gatan Bioscan Camera, Model 792).

### Energy dispersive spectroscopy (EDS) analysis

TEM sections of malarial hemozoin, including the heme aggregation sphere (HAS) and crystal-like hemozoin, were analyzed using energy dispersive (X-ray) spectroscopy mapping by Oxford INCA software (Lyford, UK) under a JEM-2010 transmission electron microscope at an accelerating voltage of 200 kV, as previously described [31]. The Oxford INCA software package was used to carry out the X-ray analysis to determine the elemental composition of the sample and generate its characteristic spectrum.

### Analysis of the effect of artemether on malarial parasites using light microscope

BALB/c or ICR mice were infected via intraperitoneal injections with *P. yoelii* 17XNL. When the infection rate reached 15–20%, the mice were treated orally with artemether at a dose of 100 mg/kg. Blood samples were collected 4 and 16 h post-treatment. Blood smears were prepared and stained with Giemsa according to standard staining protocols, and the smears were subsequently examined under a light microscope (Nikon 50i).

### Comparison of hemozoin content and merozoite number

*Plasmodium falciparum* 3D7^WT^ and *P. falciparum* 3D7^C580Y^ were purified using a 40–70% Percoll gradient and subsequently treated with 5% sorbitol to obtain 6-h ring stages. The cultures were then incubated for 36–42 h, adjusting the parasitemia to 0.5–1%. Blood smears were prepared and stained with Giemsa according to standard staining protocols, followed by a comparison of the merozoite counts using a light microscope (Motic, PA53 FS6). Approximately 10 mL of the culture medium was treated with a 0.15% saponin solution on ice for 10 min to lyse the cells. After centrifugation at 10,000 *g* for 15 min, the pellet was washed with 25 mmol/L Tris (pH 7.8) containing 2.5% sodium dodecyl sulfate until the supernatant was clear. The pellet was then dissolved in 250 µL of 2.5% sodium dodecyl sulfate buffer and 20 µL of 2.5 mol/L NaOH. The absorbance of hemozoin was measured at 400 nm using a Nanodrop 2000 spectrophotometer for content quantification, as described in a previous study [35]. Statistical analysis was performed using an unpaired *T*-test with GraphPad Prism 8.0.2 software for Windows, considering a *p*-value of less than 0.05 as statistically significant.

### Transcriptome analysis of *P. falciparum* 3D7

The *P. falciparum* 3D7 strain was cultured in RPMI 1640 medium following established protocols [34, 35]. The parasites were synchronized using 5% sorbitol, and mature schizonts were subsequently purified on a 40–70% Percoll gradient. Cultures were then treated with 5% sorbitol to obtain ring stages at 0–3 h post-invasion (hpi). Parasites were collected within this time frame, and samples were harvested at 0, 6, 12, 18, 24, 30, 36, and 42 hpi for transcriptome analysis. Samples at different stages were collected and preserved in TRIzol (Invitrogen, USA). RNA extraction was performed using phenol and isopropanol precipitation. RNA quantification and quality assessment were conducted using a Nanodrop 2000 (Thermo Fisher Scientific, Waltham, MA, USA), while RNA integrity was evaluated using an Agilent 2100 Bioanalyzer (Agilent Technologies, Santa Clara, CA, USA). Libraries were prepared using a TruSeq Stranded mRNA LT Sample Prep Kit (Illumina, San Diego, CA, USA). Transcriptome sequencing and analysis were performed by LC Sciences (Hangzhou, Zhejiang, China).

## Results

### The hemozoin granules were degraded and utilized in *Schistosoma japonicum* gut

**Fig. 1:**
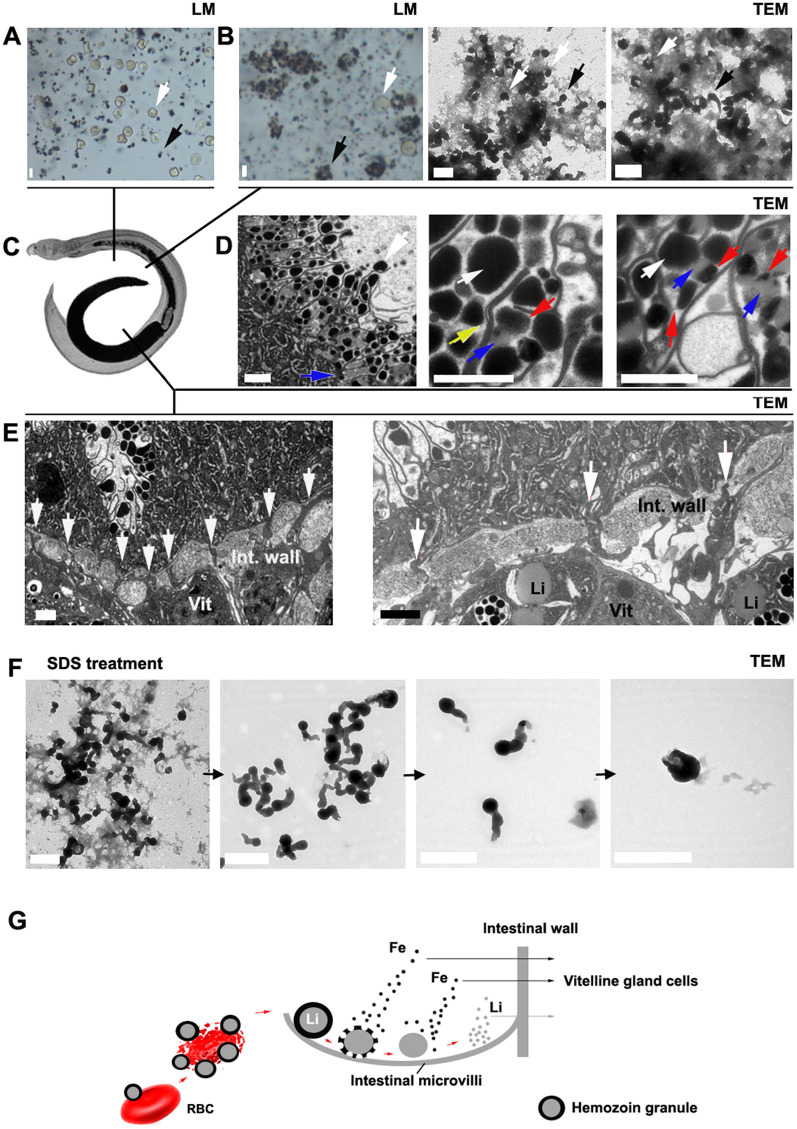
Morphology and degradation of *Schistosoma* hemozoin granules. **A** When *Schistosoma japonicum* ingests host red blood cells, these cells mix with hemozoin granules in the anterior portion of the gut, where the majority remain intact and are not associated with the granules. The white arrow indicates an intact erythrocyte, while the black arrow shows an erythrocyte that is either adsorbed by hemozoin granules or utilized in the formation of new granules. **B** Light microscopy (LM) images demonstrate that these red blood cells are either adsorbed by or utilized in the formation of hemozoin granules. Transmission electron microscopy (TEM) images reveal that hemozoin granules incorporate degraded red blood cells to form new granules. The black arrow points to a comma-shaped granule, while the white arrows indicate globe-shaped granules. **C** A *Schistosoma japonicum* specimen indicates the observation position. **D** The degradation of hemozoin granules occurs near the microvilli of the intestinal wall in the posterior region of the *Schistosoma* gut. The white arrows denote the hemozoin granules, red arrows indicate the outer heme polymer layer of the degrading hemozoin granules, blue arrows point to the inner lipid layer of the hemozoin granules, and the yellow arrow shows the microvilli with cavities that store and secrete materials to decompose hemozoin granules. **E** TEM images illustrate that intestinal contents are transported to the vitelline gland cells across the intestinal wall. The white arrows indicate the transport locations across the intestinal wall. Li, lipids; Vit, vitelline gland cells; Int. wall, intestinal wall. **F** TEM images display the morphologies of *S. japonicum* hemozoin granules following treatment with SDS solution. After globe- or comma-shaped granules are treated with 1–2.5% SDS for 0.5–1 h, the matrix is initially removed, followed by the disappearance of the lipid-like tails of the comma-shaped granules with prolonged exposure. **G** The schematic diagram illustrates the process by which a hemozoin granule attaches to an erythrocyte, subsequently utilizing the erythrocyte to form additional hemozoin granules, which are then degraded near the intestinal microvilli of the *Schistosoma* hemozoin, accompanied by the transport of iron and lipids across the intestinal wall into the vitelline gland cells. All scale bars represent 1 μm

The anterior portion of the intestinal tract of *Schistosoma japonicum* contains globe- and comma-shaped hemozoin granules, along with freshly ingested host red blood cells (Fig. 1A–C). Examination using light microscopy (LM) and transmission electron microscopy (TEM) reveals that the red blood cells are surrounded by black hemozoin granules, forming clusters or aggregates. Our previous studies indicate that upon attachment to erythrocytes, *Schistosoma* hemozoin granules can utilize these cells for the self-assembly of new hemozoin granules [32]. Under TEM, hemozoin granules are observed to be in close contact with the matrix derived from degraded erythrocytes (Fig. 1B). During the formation of hemozoin granules, heme aggregates on the surface of a lipid droplet, as previously observed [32, 36, 37], ultimately resulting in the formation of comma-shaped granules [32]. Notably, in the posterior portion of the intestine, hemozoin granules are degraded near the intestinal microvilli (Fig. 1C, D). Initially, the heme polymer in the outer layer of the hemozoin granule decomposes, followed by the subsequent degradation of the lipid components within the granules (Fig. 1D). Since *Schistosoma* hemozoin granules are composed of heme polymer and lipids, degradation can release heme, iron, and lipids. In vitro experiments demonstrated that under SDS treatment, these globe- and comma-shaped hemozoin granules lost their lipid matrix and lipid-like tails, leaving behind the head portion, which consists of heme polymer (Fig. 1F). Thus, after the degradation of these granules near the intestinal microvilli, a significant number of lipid droplets can be observed (Fig. 1D). Following hemozoin degradation, the transport of intestinal contents across the intestinal wall to vitelline gland cells becomes visible under TEM (Fig. 1E). Simultaneously, lipid accumulation is detected within the vitelline gland cells (Fig. 1E). On the basis of these observations, we propose that hemozoin granules in schistosomes facilitate the transport of heme and lipids by forming and subsequently degrading (Fig. 1G). Clearly, *Schistosoma* hemozoin granules play a crucial role in storing and transporting heme, iron, and lipids during the development and reproduction of *Schistosoma*.

### Morphology of malarial hemozoin differs from that of *Schistosoma* hemozoin granules

**Fig. 2:**
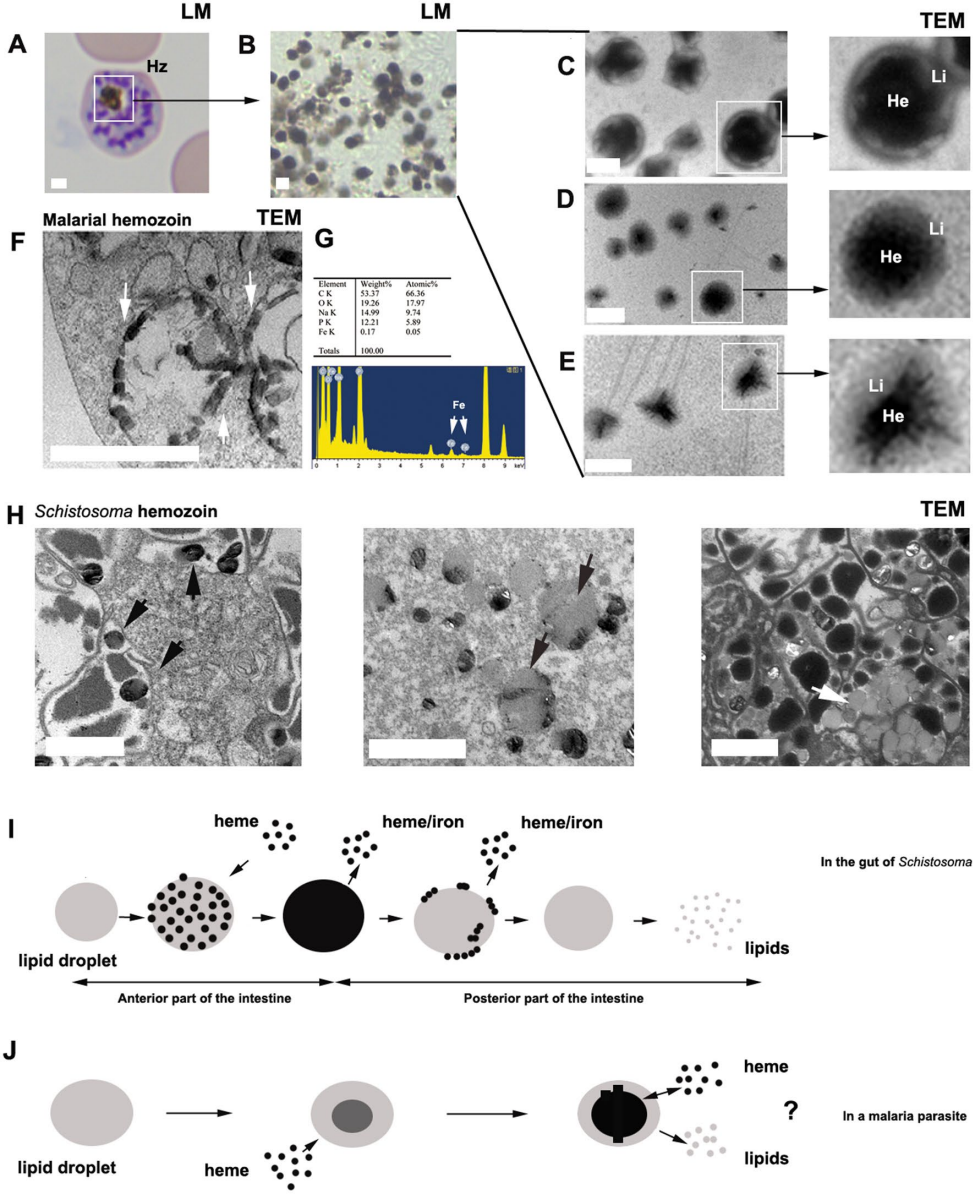
Comparison of malaria hemozoin and *Schistosoma* hemozoin. **A** Under light microscopy (LM), malaria hemozoin is observed within a schizont. Hz, hemozoin. **B** Freshly collected malaria hemozoin as visualized under LM. **C**–**E** Collected malaria hemozoin examined using transmission electron microscopy (TEM). He, heme polymer or heme aggregation; Li, lipids. **F** Formation of malaria hemozoin in the digestive vacuole along with the membrane. White arrows indicate hemozoin. **G** Energy spectrum analysis reveals the presence of iron in malaria hemozoin. **H** Various degrees of degraded *Schistosoma* hemozoin granules are located near the microvilli in the *Schistosoma* gut. Black arrows indicate the degraded granules, while the white arrow indicates the lipid droplets after the degradation of the heme polymer layer of the hemozoin granules. **I** This schematic diagram illustrates that in the anterior section of the *Schistosoma* intestine, free heme accumulates in lipid droplets, resulting in the formation of hemozoin granules. Subsequently, in the posterior region of the gut, these granules undergo degradation, beginning with the heme polymer layer, followed by the lipid layer. **J** In malaria parasites, free heme dissolves in lipid droplets, initially forming aggregates or polymers that can develop into crystal-like structures. The combination of heme polymers and lipids constitutes hemozoin, which may exist in a dynamic equilibrium with the cytoplasm, facilitating the absorption and release of heme and lipids. All scale bars represent 1 μm

Under light microscopy (LM), these dark-brown malarial hemozoin appears either globular or irregular in shape (Fig. 2A, B). Transmission electron microscopy (TEM) reveals various morphologies of malarial hemozoin (Fig. 2C–E). Energy dispersive spectroscopy can detect iron within these hemozoin, as previously studied [38] (Fig. 2G). When observed under TEM, hemozoin is identified as a mixture of heme polymer and lipids. In the digestive vacuole of malarial parasites, hemozoin is seen to form along the membrane of the digestive vacuole (Fig. 2F), consistent with previous reports [39–42]. However, in malaria parasites, the degradation of hemozoin is not easily observed. In contrast, *Schistosoma* hemozoin granules exhibit gradual degradation from the outer hemozoin layer to the inner lipid layer, which occurs adjacent to the intestinal microvilli (Fig. 2H). On the basis of the morphology of malarial hemozoin, it is likely that during its formation, free heme dissolves in lipid droplets, facilitating the polymerization of heme into a crystal-like structure. Some researchers consider only the crystal-like structure as hemozoin, overlooking its matrix. Upon observing fresh samples with TEM, we found that malaria hemozoin is a combination of heme aggregation or crystal-like heme polymer and lipids, where heme dissolves in the lipid-like matrix, gradually forming hemozoin crystal (Fig. 2C–E). It is evident that the morphology and structure of malarial hemozoin differ from those of *Schistosoma* hemozoin granules. This difference may be attributed to their distinct functions in *Schistosoma* and *Plasmodium*, or the manner in which they execute these functions (Fig. 2I, J).

For *Schistosoma* hemozoin, formation occurs in the anterior region of the *Schistosoma* gut, and is subsequently degraded in the posterior region to release iron and lipids, which are then transported to the vitelline gland cells and eggs (Fig. 2H, I), as previously described [31]. In contrast, the degradation of hemozoin in malaria parasites is not observed, unlike that in *Schistosoma* hemozoin granules. It is perplexing that if malarial hemozoin is indeed a waste product, it should be excreted from the cell upon formation; however, we have never observed such excretion. Alternatively, malarial hemozoin may serve an unidentified function.

### The association between malaria hemozoin formation and parasites

**Fig. 3:**
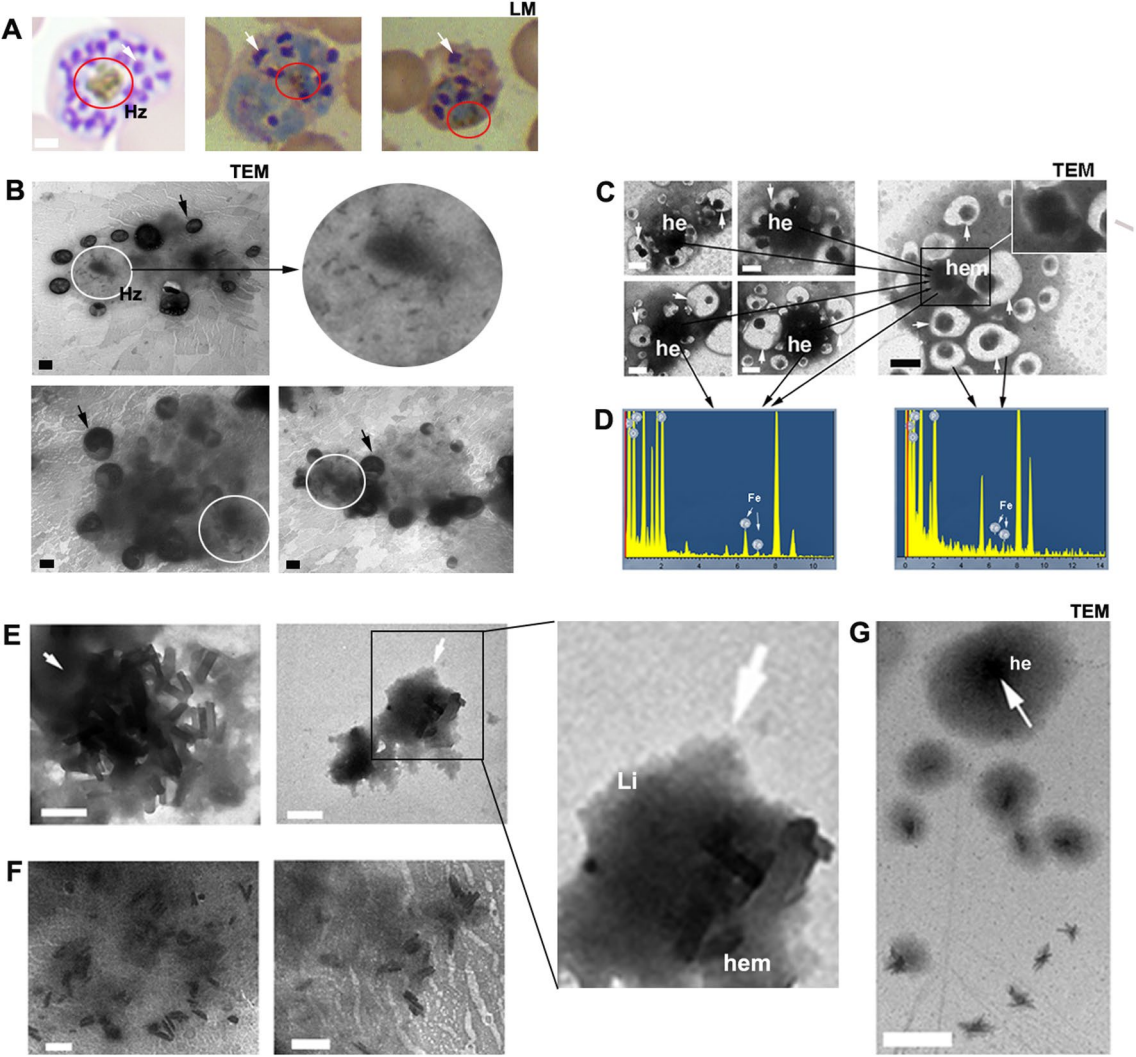
Morphology of hemozoin in schizonts. **A** Malaria hemozoin (indicated by the red circle) in schizonts, as observed under light microscopy (LM). The white arrow points to the merozoite. **B** Malaria hemozoin (indicated by the white circle) in schizonts, visualized using transmission electron microscopy (TEM). The black arrow indicates the merozoite. **C** Under TEM, hemozoin in schizonts appears as black cotton, with occasional detection of crystal-like structures. **D** Energy spectrum analysis reveals that this black cotton contains iron, while iron is not detected in merozoites (indicated by white arrows). **E** Under TEM, hemozoin is observed as a combination of heme polymers and lipids, with the crystal-like heme polymer formed from the lipid matrix. **F** Upon treatment with sodium dodecyl sulfate (SDS), only the crystal-like structure remains. **G** During hemozoin formation, heme initially accumulates within a lipid droplet, resulting in the formation of a heme aggregation sphere, which appears as a black cotton-like structure in schizonts. Hz, hemozoin; Li, lipid; hem, crystal-like heme polymer; he, heme aggregation sphere. All scale bars indicate 1 μm

Under light microscopy (LM), malaria hemozoin at various developmental stages can be distinctly observed. Notably, during the *Plasmodium* schizont stage, hemozoin appears larger and more prominent compared with other stages (Fig. 3A). In addition, under transmission electron microscopy (TEM), hemozoin within a schizont is observed as a combination of crystal-like structures and lipid spheres, distributed near merozoites (Fig. 3B). When fresh samples are examined using TEM, a mass of black, cotton-like structures is frequently identified (Fig. 3C, G). Occasionally, a crystal-like structure can be detected within the “black cotton” of a parasite (Fig. 3C). Energy spectrum analysis reveals that the “black cotton” contains iron, which is not detected in the merozoites. Importantly, hemozoin, comprising crystal-like structures and lipids, indicates that the crystal-like structures form from the lipid matrix (Fig. 3E). The crystal-like structures and lipid matrix are interdependent; when the lipid matrix is removed using sodium dodecyl sulfate (SDS), incomplete crystal-like structures can be observed (Fig. 3F). This observation is similar to the *Schistosoma* hemozoin granules.

In the digestive vacuole, the ingested host lipids contribute to the accumulation of heme derived from hemoglobin decomposition. Furthermore, the polymerization of heme necessarily involves electron transfer between heme polymers and lipids, resulting in lipid remodeling. Given that the modified host lipids are crucial for parasites, and are closely associated with hemozoin formation, this provides insight into why hemozoin is not expelled from parasites.

### Relationship between heme or iron utilization and hemozoin formation during the erythrocytic stage

**Fig. 4:**
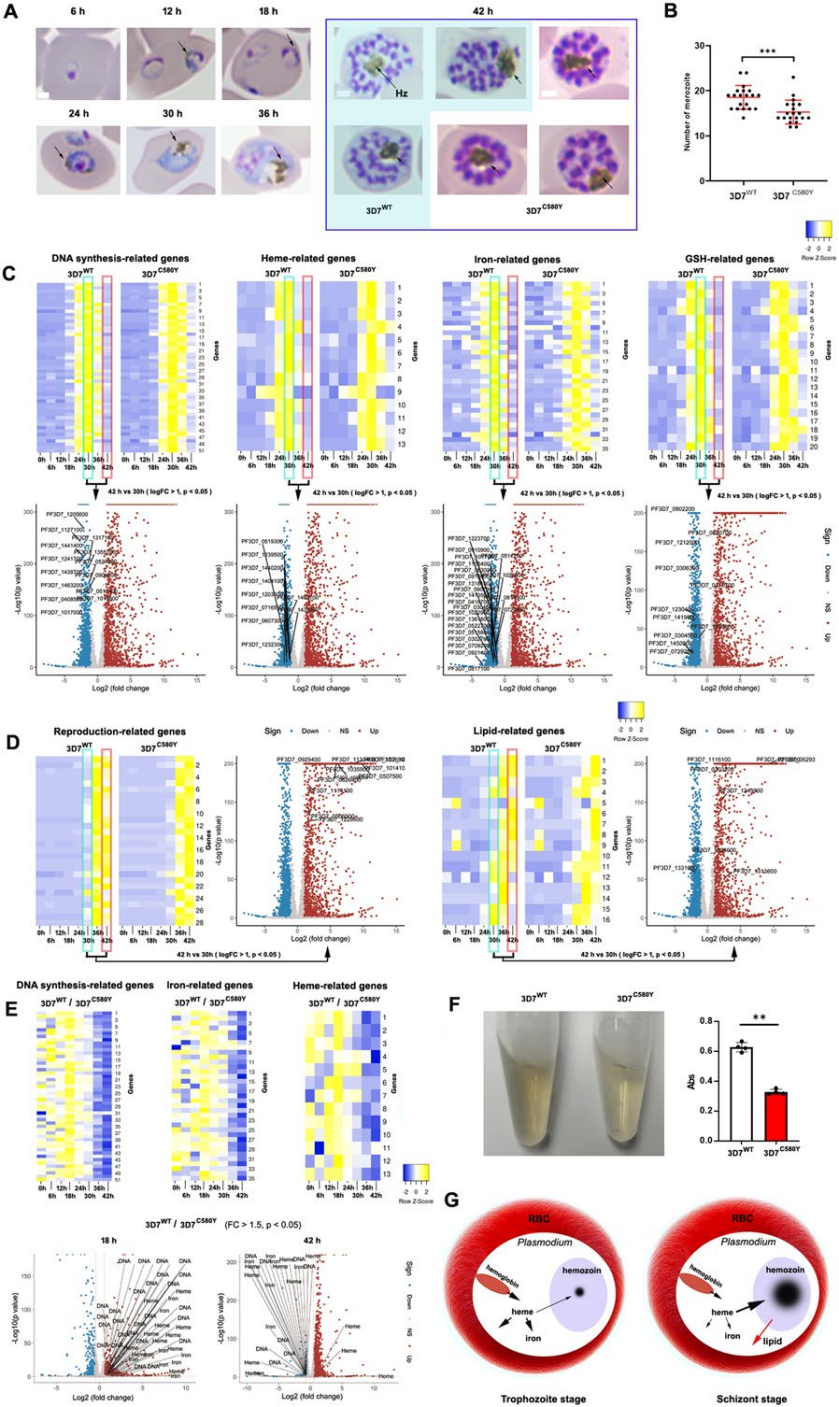
Relationship between heme or iron utilization and hemozoin formation. **A** Formation of malaria hemozoin at 6, 12, 18, 24, 30, 36, and 42 h post-infection with *P. falciparum*. **B** Comparison of merozoite numbers between *P. falciparum* 3D7^WT^ and *P. falciparum* 3D7^C580Y^ reveals that *P. falciparum* 3D7^C580Y^ produces fewer merozoites compared with *P. falciparum* 3D7^WT^. **C** Gene expression heatmaps display expression patterns across various developmental stages of *P. falciparum* 3D7^WT^ and *P. falciparum* 3D7^C580Y^. Volcano plots illustrate differential gene expression between 30 and 42 h post-infection. Supplementary Data 1–4 provide detailed information on gene expression. **D** Heatmaps of gene expression indicate the expression patterns of reproduction- and lipid-related genes. Volcano plots show differential gene expression between 30 and 42 h post-infection (see Supplementary Data 5–6). **E** Differential expression analysis of genes associated with DNA synthesis, iron, and heme utilization was conducted at 18 h and 42 h between *P. falciparum* 3D7^WT^ and *P. falciparum* 3D7^C580Y^. **F** Comparison of hemozoin content between *P. falciparum* 3D7^WT^ and *P. falciparum* 3D7^C580Y^ indicates that *P. falciparum* 3D7^C580Y^ produces less hemozoin than *P. falciparum* 3D7^WT^. **G** The schematic diagram illustrates that during the trophozoite stage, parasites consume more heme and iron, resulting in reduced heme accumulation, and consequently, less hemozoin formation. Conversely, in the schizont stage, parasites consume lower amounts of heme and iron, leading to increased heme accumulation and greater production of hemozoin. Furthermore, the interaction between hemozoin formation and host lipids suggests that increased hemozoin production can supply more processed lipids for parasite utilization. Hz, hemozoin. All scale bars indicate 1 μm. Asterisks denote significance: **p < 0.01; ***p < 0.001

The formation of hemozoin is observed early, with faintly visible dark-brown particles detected in parasites at 12 h post-infection. By 30 h post-infection, noticeable hemozoin appears in the parasite (Fig. 4A). Notably, at 42 h post-infection, hemozoin is more prominent and larger in the mature schizont (Fig. 4A). It is intriguing to understand why such a significant amount of hemozoin is produced in the mature schizont.

Since hemozoin formation is associated with hemoglobin digestion, heme release, and iron storage, we analyzed the expression of related genes to elucidate the relationship between hemozoin formation and the utilization of heme or iron. Our analysis of gene expression at different developmental stages revealed that genes related to iron, heme, DNA synthesis, and GSH exhibited predominant expression at 30 h post-infection. Concurrently, hemozoin accumulation increased alongside cytoplasmic content during this time (Fig. 4A, C). This suggests that as DNA begins to replicate, the demand for iron rises, thereby enhancing the GSH cycle to facilitate heme degradation [43]. Notably, the expression of these genes was not upregulated at 42 h post-infection (Fig. 4C), indicating a reduced function related to DNA synthesis and heme and iron utilization at this stage. In contrast, genes associated with reproduction and lipid utilization were upregulated at this time (Fig. 4D), suggesting that at 42 h post-infection, the schizont prioritizes the production of merozoites over the utilization of heme or iron, as well as the continuation of DNA synthesis.

To investigate the effect of reduced hemoglobin digestion and heme release on hemozoin formation, we compared gene expression and hemozoin formation between *P. falciparum* 3D7^WT^ and *P. falciparum* 3D7^C580Y^. Given that mutations in the *P. falciparum* Kelch 13 protein (*Pf*K13) dampen hemoglobin endocytosis [44, 45], parasites with these mutations inevitably exhibit reduced iron and heme utilization. We found that the number of merozoites and the total hemozoin content of *P. falciparum* 3D7^C580Y^ were lower than those of *P. falciparum* 3D7^WT^ (Fig. 4B, F). These results suggest that decreased hemoglobin endocytosis and heme release reduce hemozoin production and its reproduction, thereby confirming the correlation between low heme utilization and diminished hemozoin formation [35]. However, no significant changes were observed in the chronological expression of genes related to DNA synthesis, GSH, reproduction, lipid metabolism, and heme and iron utilization (Fig. 4C).

Notably, both *P. falciparum* 3D7^WT^ and *P. falciparum* 3D7C^580Y^ downregulated gene expression related to DNA synthesis, glutathione (GSH), heme, and iron utilization at the 42-h post-infection stage (Fig. 4C), indicating that the reduction in heme and iron utilization at this time point is not affected by diminished hemoglobin supply. Furthermore, the expression levels in *P. falciparum* 3D7^WT^ were slightly lower than those in *P. falciparum* 3D7^C580Y^ (Fig. 4E), likely suggesting that *P. falciparum* 3D7^WT^, with normal iron and heme supply, decreases their utilization to a greater extent. The reduced expression of genes associated with heme and iron utilization indicates decreased utilization, which may lead to the accumulation of heme for hemozoin formation. Hemozoin formation is linked to lipid remodeling, and the increased remodeling of host lipids is crucial for merozoite production. In addition, genes related to lipid metabolism and reproduction were upregulated at this stage. These findings suggest that the specific downregulation of iron- and heme-related genes results in heme accumulation and hemozoin formation, likely facilitating the remodeling of host lipids and subsequently promoting merozoite production (Fig. 4G).

### What happens when hemozoin is released from parasites?

**Fig. 5:**
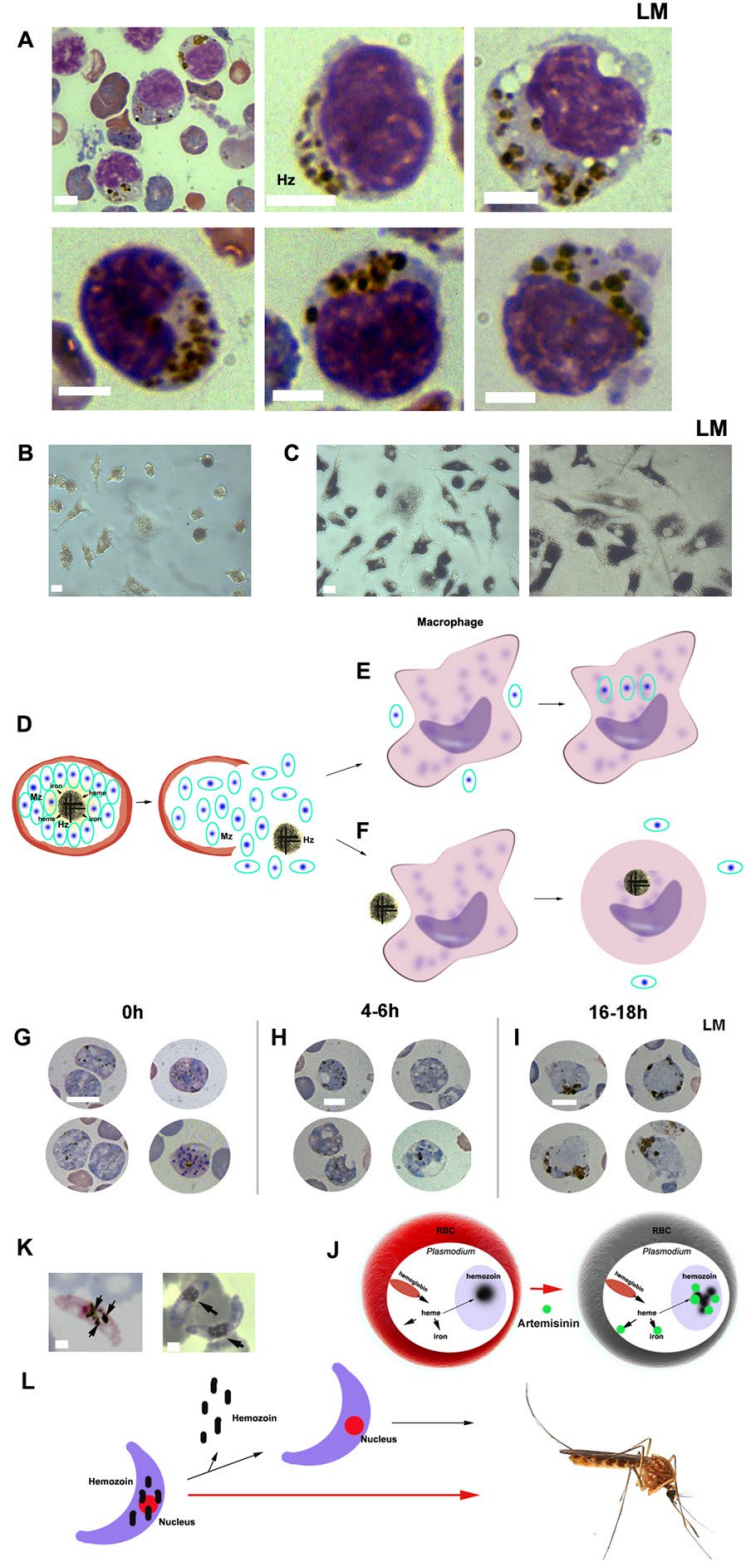
Effect of hemozoin on macrophages and *Plasmodium*. **A** A large number of mononuclear macrophages that uptake hemozoin were observed in infected mice. **B**, **C** The macrophages sustain damage when they phagocytose significant amounts of hemozoin in vitro (**C**), compared with the normal group (**B**). **D**–**F** This schematic diagram illustrates that when schizonts release merozoites, host macrophages can ingest and eliminate the merozoites (**E**); however, after macrophages ingest hemozoin, they are unable to eliminate the merozoites owing to damage or death caused by the ingested hemozoin (**F**). **G**–**I** Effect of artemether on *P. yoelii* 17XNL. Compared with the parasites observed at 0 h post-artemether treatment, vacuoles appear in the cytoplasm of the parasites at 4–6 h post-treatment. In contrast, at 16–18 h post-treatment, the cells become disrupted, and hemozoin aggregates abnormally. **J** This schematic diagram illustrates the mechanism through which artemisinin induces abnormal aggregation of hemozoin. Malaria parasites ingest hemoglobin to release heme, which is then degraded to release iron for the parasite’s metabolic needs. Excess heme is sequestered as hemozoin, thereby maintaining a balance between heme, iron, and their utilization within the parasite. In the presence of artemisinin, it interacts with heme or hemozoin, resulting in abnormal aggregation and disrupting the established balance, ultimately leading to parasite death. **K** A substantial amount of hemozoin (indicated by black arrows) can be detected in gametocytes under light microscopy. **L** This schematic diagram raises the question of whether hemozoin, as a waste product, should be expelled by gametocytes before entering the mosquito; however, they actually transport hemozoin into the mosquito. Hz, hemozoin; Mz, merozoite. All scale bars indicate 5 μm

When a schizont ruptures, hemozoin and merozoites are released into the host’s circulation. It has been consistently observed that mononuclear macrophages engulf substantial amounts of hemozoin (Fig. 5A), a phenomenon reported by numerous researchers [46]. Furthermore, in vitro experiments have demonstrated that when a macrophage engulfs excessive amounts of hemozoin, it may become deformed or even rupture (Fig. 5B, C). Clearly, the release of a significant quantity of hemozoin into the circulation is likely to disrupt macrophage functions, creating conditions that facilitate the survival and infection of more schizonts in red blood cells (Fig. 5D–F).

### Artemether disrupts heme utilization, leading to parasite death and highlighting the importance of heme and hemozoin

Recent research indicates that artemisinin can form adducts with heme, disrupting heme utilization and leading to parasite death [47]. Clearly, the sequestration of heme impacts the formation of hemozoin. To investigate the interaction between artemisinin and hemozoin, we administered artemether to mice infected with *P. yoelii* 17XNL at a dose of 100 mg/kg. Cytoplasmic vacuolation was observed 4–6 h post-treatment with artemether. After 16–18 h of treatment, we noted a significant accumulation of abnormal hemozoin, accompanied by the destruction of normal cell structures (Fig. 5G, I). Notably, recent studies suggest that parasite death results from heme depletion and hemozoin damage induced by artemether [47]. This implies that heme and hemozoin formation are crucial for parasite survival; if heme utilization or the interaction between heme and hemozoin is disrupted, the parasite will perish (Fig. 5J). It is likely that the balance between heme and hemozoin is essential for maintaining adequate heme levels within parasites. Furthermore, the established role of *Schistosoma* hemozoin supports this hypothesis [31, 32].

## Discussion

Hemozoin is commonly regarded as a waste product resulting from the detoxification of free heme. However, in *Schistosoma*, hemozoin granules are formed in the anterior portion of the gut and subsequently degraded in the posterior section [31]. During this process, iron is observed to transfer from erythrocytes to hemozoin granules and then to vitelline gland cells through the intestinal wall, ultimately reaching the eggs [31, 32]. Concurrently, lipids, another component of hemozoin granules, are also degraded and accumulate in vitelline gland cells. These findings indicate that the formation and degradation of hemozoin granules facilitate the transfer of heme, iron, and lipids from erythrocytes to vitelline gland cells and eggs in *Schistosoma*. Thus, the function of hemozoin extends beyond mere waste disposal; it serves as a crucial medium for iron storage and transport, playing a pivotal role in the reproduction of *Schistosoma*.

In contrast to multicellular organisms, malaria parasites, which belong to the class Protozoa, exhibit distinct characteristics. The direct observation of the relationship between hemozoin and the transport of iron, heme, or lipids within a cell is not feasible. However, various phenomena provide valuable insights, indicating that malarial hemozoin is not merely a waste product. For instance, hemozoin is exclusively released from the parasite during schizont rupture, rather than at any stage of its erythrocytic cycle. In addition, hemozoin is transmitted to the next host along with gametocytes. The development and survival of *Plasmodium* parasites heavily depend on a substantial amount of iron, as evidenced by their sensitivity to iron chelators. Paradoxically, despite their reliance on a stable and high level of heme throughout their development within red blood cells [16], malarial parasites lack the proteins or mechanisms necessary to store iron or heme [48, 49]. Therefore, establishing a mechanism to store sufficient iron and heme to meet the demands of the parasites is crucial. Hemozoin, as a polymer of iron and heme, may fulfill this requirement. In fact, when malaria parasites reduce their iron and heme requirements, their hemozoin content also decreases correspondingly, suggesting a close association between heme utilization and hemozoin formation.

Through the analysis of gene expression, we determined that genes associated with heme, iron utilization, and Glutathione (GSH) are predominantly expressed 30 h post-infection. Glutathione can degrade heme to release iron in *Plasmodium* [50–53]. Moreover, genes related to DNA synthesis are also expressed at this stage. These results suggest that a significant release and utilization of iron at this stage can enhance DNA synthesis [54–57]. Hemozoin accumulation increases at this stage, although its content remains lower than that observed in later stages. At 42 h post-infection, genes related to iron and heme utilization are downregulated, suggesting a decreased reliance on these elements. This likely leads to the accumulation of heme and the formation of substantial amounts of hemozoin, the significance of which for the parasites has remained unclear. Recent studies propose that artemisinin exerts its antiparasitic effects by disrupting heme utilization and altering hemozoin structure [47]. In this experiment, we treated mice infected with *P. yoelii* 17XNL using artemether and observed dead parasites exhibiting abnormal hemozoin accumulation. Previous studies also indicate that artemisinin interacts with heme to inhibit hemozoin crystallization and heme detoxification [58]. In conjunction with recent research demonstrating that artemisinin can form adducts with heme, thereby disrupting heme utilization and leading to parasite death [47], it is evident that heme utilization and hemozoin formation are essential for parasite survival. If hemozoin was merely a waste product of detoxification, it would be expelled promptly rather than continuously stored within the cell. Therefore, it is clear that hemozoin likely plays an unknown yet crucial role in these parasites.

At 36 and 42 h post-infection, there is a significant upregulation of genes associated with reproduction and lipid utilization. This observation aligns with the requirement for substantial amounts of lipids in the production and development of merozoites. *Plasmodium* parasites depend on lipids sourced from their hosts [59], which have been identified within the acidic digestive vacuole of the parasites [60–63]. However, host lipids within the digestive vacuole cannot be utilized directly by the parasites; they must undergo processing and remodeling prior to being utilized [64–66]. Notably, hemozoin formation and lipid remodeling are interconnected processes within the digestive vacuoles of malarial parasites. Specifically, both heme polymerization and hemozoin formation depend on lipids [42, 67, 68], while the completion of lipid oxidation and remodeling also necessitates hemozoin formation [69, 70]. Therefore, hemozoin formation not only serves as a detoxification process but also plays a crucial role in the utilization of host lipids, particularly considering the limited capacity of malaria parasites to synthesize fatty acids or lipids. During the schizont stage, when numerous merozoites develop and mature, hemozoin crystals are abundantly formed. Unsaturated fatty acids can enhance hemozoin formation [29]. As unsaturated fatty acids are converted into saturated fatty acids through hemozoin formation, host-derived fatty acids or lipids undergo remodeling. The substantial formation of hemozoin during the schizont stage suggests that extensive remodeling and utilization of lipids contribute to the development of merozoites and their membranes. Furthermore, this observation is consistent with previous studies indicating an increase in saturated fatty acids during the schizont stage [71]. In addition, the high expression levels of lipid-related genes at this stage further corroborate these observed changes and trends. Transmission electron microscopy (TEM) has revealed that hemozoin consists of a lipid matrix combined with a crystal-like structure. Moreover, hemozoin formation is observed to occur near the developing merozoites. Although hemozoin is generally regarded as a waste product, parasites do not expel it, likely involving lipid utilization throughout their development.

It is noteworthy that, in addition to supporting parasite development and reproduction, the released hemozoin is also likely to significantly contribute to parasite protection. When a substantial amount of hemozoin is released into circulation and ingested by mononuclear macrophages, it impairs the phagocytosis performed by these immune cells in the host [70, 72]. Furthermore, hemozoin has been found to inhibit the differentiation and maturation of human monocyte-derived dendritic cells [73], as well as impair the chemotactic motility and transendothelial migration of monocytes [74]. Consequently, hemozoin impairs the host’s immunity response, providing protection for parasites and their merozoites. In addition, a large amount of hemozoin was carried to the mosquito host by gametocytes (Fig. 5K, L). If hemozoin was merely a waste product, it would be perplexing why gametocytes, which carry this waste, enter the mosquito. Furthermore, malaria parasites exclusively initiate heme synthesis in mosquitoes [24, 25]. It is important to note that hemozoin is a polymer of heme. In *Schistosoma* hemozoin, hemozoin functions as a carrier for heme or iron, facilitating the transport of these elements within the organism. These observations suggest that hemozoin in malaria parasites likely serves as a heme carrier, fulfilling the heme requirements during the transition between the two hosts.

In essence, hemozoin may play a crucial role throughout the entire life cycle of *Plasmodium*, including growth, development, and reproduction, potentially providing protection for parasites and merozoites against elimination by macrophages. Although malarial hemozoin is functionally similar to *Schistosoma* hemozoin, their mechanisms of action differ slightly. *Schistosoma* hemozoin transports iron and lipids from erythrocytes to vitelline glands, and ultimately to eggs, through its formation and degradation [31, 32]. In contrast, malarial hemozoin likely adjusts heme or iron balance in parasites, and may remodel host lipids to meet its requirements. In addition, hemozoin in gametocytes may store heme or iron for development in mosquitoes. Although further study is needed to elucidate the roles of hemozoin in parasites and associated diseases, it is evident that hemozoin should not be merely considered a waste product of heme detoxification.

## Supplementary Information

Supplementary Material 1. Supplementary files for differential gene expression analysis from 0 to 42 h post-infection across 8 stages.

Supplementary Material 2. Data 1. DNA synthesis-related genes

Supplementary Material 3. Data 2. Heme-related genes

Supplementary Material 4. Data 3. Glutathione-related genes

Supplementary Material 5. Data 4. Iron-related genes

Supplementary Material 6. Data 5. Reproduction-related genes

Supplementary Material 7. Data 6. Lipid-related genes

## Data Availability

No datasets were generated or analyzed during the current study.
